# Identification and characterization of novel B-cell epitopes within EBV latent membrane protein 2 (LMP2)

**Published:** 2011-01-10

**Authors:** Xiangyang Xue, Shanli Zhu, Wenshu Li, Jun Chen, Qin Ou, Meixia Zheng, Wenci Gong, Lifang Zhang

**Affiliations:** 1 Department of Microbiology and Immunology, Wenzhou Medical College, Wenzhou, Zhejiang 325035, PR China

## 

The purpose of this study was to screen and identify the linear B cell epitopes of Epstein-Barr virus (EBV) latent membrane protein 2 (LMP2). The secondary structure and the surface properties of EBV LMP2A protein were analyzed. Then, the peptides with good hydrophilicity, high accessibility and flexibility and strong antigenicity were chosen and average antigenicity index (AI) of epitope peptide was further investigated. Three peptides were selected as potential linear B cell epitopes. The location and the sequence of amino acid were 199-209 (RIEDPPFNSLL), 318-322 (TLNLT) and 381-391 (KSLSSTEFIPN), respectively. The genes encoding potential B cell epitope were cloned and expressed in *E. coli* system. The immune sera of above different purified fusion proteins were obtained from BLAB/c mice by subcutaneously immunization for three times. Western blot showed that these epitope recombinant proteins could be recognized by the serum antibodies against the whole LMP2 of EBV obtained from nasopharyngeal carcinoma (NPC). Indirect ELISA measured the reactivity of individual sera from 196 NPC patients, 44 infectious mononucleosis (IM) and 108 healthy individuals to these epitope-fused proteins indicated that NPC patients were significantly higher compared with IM and healthy individuals (*P*<0.05). In addition, all the immune sera of peptide-fused proteins could response to native LMP2A antigen obtained from the EBV prototype strain, B95-8 cells. IFA confirmed that the recognition of the specific antibodies induced by the immune sera of epitope peptide-fused proteins was intracellular regions of LMP2A. These results demonstrated that these three predictive epitopes not only were immunodominant B-cell epitopes of LMP2A, but also may be potential targets for application in the design of diagnostic tools.

